# Serum zinc deficiency and progression of cardiovascular- kidney-metabolic syndrome

**DOI:** 10.3389/fnut.2026.1910233

**Published:** 2026-07-23

**Authors:** Kuan-Chieh Tu, Mei-Yuan Liu, Kuo-Chuan Hung, Yi-Chen Lai, Chih-Cheng Lai, Yu-Min Lin, Jheng-Yan Wu

**Affiliations:** 1 Division of Cardiology, Department of Internal Medicine, Chi Mei Hospital, Chiali, Taiwan; 2 Department of Nutrition, Chi Mei Medical Center, Tainan, Taiwan; 3 Department of Nutrition and Health Sciences, Chang Jung Christian University, Tainan, Taiwan; 4 Department of Hospitality Management, Southern Taiwan University of Science and Technology, Tainan, Taiwan; 5 Department of Anesthesiology, Chi Mei Medical Center, Tainan, Taiwan; 6 School of Medicine, College of Medicine, National Sun Yat-sen University, Kaohsiung, Taiwan; 7 Department of Intensive Care Medicine, Chi Mei Medical Center, Tainan, Taiwan; 8 Division of Cardiology, Department of Internal Medicine, Chi Mei Medical Center, Tainan, Taiwan; 9 Department of Public Health, College of Medicine, National Cheng Kung University, Tainan, Taiwan

**Keywords:** cardiovascular disease, cardiovascular-kidney-metabolic syndrome, chronic kidney disease, CKM stage progression, serum zinc deficiency

## Abstract

**Introduction:**

Cardiovascular-kidney-metabolic (CKM) syndrome represents an interconnected disease continuum linking metabolic dysfunction, chronic kidney disease (CKD), and cardiovascular disease. Serum zinc deficiency (SZD) has been associated with adverse cardiovascular and renal outcomes; however, its association with CKM progression remains unclear.

**Methods:**

This retrospective cohort study was conducted using the TriNetX. Adults with CKM stage 1 or 2 were enrolled in the study. SZD was defined as a serum zinc concentration < 70 μg/dL, while individuals with zinc concentrations between 70 and 120 μg/dL served as controls. After 1:1 matching, 74,245 patients were included in each group. The primary outcome was operational progression from CKM stage 1–2 to stage 3–4. Secondary outcomes included major adverse cardiovascular events, coronary artery disease, peripheral artery disease, atrial fibrillation, ischemic stroke, heart failure, advanced CKD, and all-cause mortality.

**Results:**

Among 148,490 matching individuals, SZD was associated with a significantly increased risk of operational progression to CKM stage 3–4 compared with normal zinc status (HR, 1.36; 95% CI, 1.30–1.41; *P* < 0.001). SZD was also associated with increased risks of other secondary outcomes (all *P* < 0.0056 after Bonferroni correction).

**Conclusion:**

Among patients with CKM stage 1–2, SZD was associated with a significantly increased risk of operational progression to CKM stage 3–4 and a broad spectrum of adverse cardiovascular, renal, and mortality outcomes. These findings suggest that SZD may represent an important marker of adverse CKM health and may help identify individuals at increased risk for CKM progression.

## Introduction

Cardiovascular-kidney-metabolic (CKM) syndrome has recently emerged as a comprehensive framework that integrates obesity, diabetes, CKD, and cardiovascular disease into a unified disease continuum (1). Rather than representing isolated conditions, these disorders interact through shared metabolic, inflammatory, and hemodynamic pathways, leading to progressive cardiovascular and renal complications. As the global burden of obesity, diabetes, and CKD continues to increase, identifying modifiable risk factors associated with CKM progression has become a major public health priority (1, 2).

Zinc is an essential trace element involved in numerous biological processes, including immune regulation, antioxidant defense, insulin signaling, endothelial function, and tissue repair (3, 4). Serum zinc deficiency (SZD) is common worldwide and is particularly prevalent among individuals with chronic diseases, malnutrition, diabetes, and CKD (5–8). Experimental studies have demonstrated that SZD promotes oxidative stress, systemic inflammation, endothelial dysfunction, and metabolic dysregulation, all of which are recognized contributors to cardiovascular and kidney disease progression (9, 10). Serum zinc concentration is the most used biomarker for assessing zinc status in clinical practice because of its widespread availability and standardized laboratory measurement (11). However, serum zinc represents only a small fraction of total body zinc and may be influenced by inflammation, acute illness, and nutritional status (12). Alternative biomarkers, such as zinc concentrations in hair or nail samples, may better reflect longer-term zinc status but are less frequently available in routine clinical settings (13).

Accumulating epidemiologic evidence has linked SZD to adverse clinical outcomes. Previous studies have reported associations between low zinc levels and increased risks of diabetes, CKD progression, cardiovascular disease, infection, frailty, and mortality. In patients with CKD, SZD has been associated with worsening renal function, protein-energy wasting, and increased hospitalization risk (14, 15). Similarly, lower zinc concentrations have been associated with a higher burden of cardiovascular risk factors and poorer cardiovascular outcomes (16, 17). Despite these observations, existing studies have largely focused on individual diseases and outcomes rather than the interconnected CKM continuum. The recently proposed CKM staging system provides an opportunity to evaluate disease progression across multiple organ systems within a unified framework. However, whether SZD is associated with progression from early-stage CKM disease to more advanced CKM stages remains unknown. Furthermore, the potential mechanisms linking SZD to CKM progression, including inflammation, atherosclerosis, and kidney dysfunction, have not been comprehensively evaluated in large-scale clinical populations. Therefore, we compared the risk of progression from CKM stage 1–2 to stage 3–4 and other cardiorenal outcomes between patients with and without SZD.

## Materials and methods

### Data source

This retrospective cohort study was conducted using the TriNetX Global Collaborative Network, a federated research network that aggregates de-identified electronic health records from 172 healthcare organizations worldwide, representing approximately 164 million individuals. The database includes demographic characteristics, diagnoses, procedures, medication prescriptions, laboratory measurements, and mortality information (18). Because only de-identified data were used, informed consent was waived. This study followed the Strengthening the Reporting of Observational Studies in Epidemiology (STROBE) reporting guideline (19).

### Study population

Adult patients (aged ≥ 18 years) with CKM stage 1 or 2 who underwent serum zinc testing within the preceding year between 1 January 2010, and 31 March 2026, were identified. CKM stages were defined according to previously proposed CKM staging criteria using diagnostic codes, medication records, and laboratory measurements. Detailed coding definitions are provided in Supplementary Table 1 (20). Patients were excluded if they had any prespecified study outcomes before the index date, lacked evidence of CKM stage 1 or 2, had missing serum zinc measurements, or had serum zinc concentrations exceeding 120 μg/dL. The index date was defined as the date on which patients first met criteria for CKM stage 1 or 2. Zinc status was classified using the most recent serum zinc measurement before the index date. Follow-up for outcomes began after the index date.

### Exposure definition

Because serum zinc measurement is the most widely available biomarker within the TriNetX database, SZD was defined as a serum zinc concentration < 70 μg/dL. Hair and nail zinc measurements, which may better reflect long-term zinc status, were not available within the database. Patients were classified into the SZD group if their serum zinc concentration was < 70 μg/dL. Individuals with serum zinc concentrations between 70 and 120 μg/dL were assigned to the control group (21, 22).

### Covariates

Baseline demographic characteristics, comorbidities, medication use, and laboratory measurements were assessed within one year before the index date. Covariates included age, sex, race, obesity, hypertension, dyslipidemia, type 2 diabetes, CKD, chronic lower respiratory diseases, liver disease, anemia, malnutrition, alcohol-related disorders, nicotine dependence, cardiovascular medications, antidiabetic medications, body mass index, estimated glomerular filtration rate (eGFR), glycated hemoglobin (HbA1c), albumin, systolic blood pressure (SBP), triglycerides, high-density lipoprotein cholesterol (HDL-C), and low-density lipoprotein cholesterol (LDL-C). Detailed coding definitions are shown in Supplementary Table 2.

### Outcomes

The primary outcome was operational progression to advanced CKM manifestations during follow-up. Because repeated longitudinal reassignment of CKM stages is not currently feasible within the TriNetX platform, this outcome was operationalized as the occurrence of incident cardiovascular disease or advanced CKD corresponding to CKM stage 3–4 manifestations among individuals with baseline CKM stage 1–2.

Secondary outcomes included major adverse cardiovascular events (MACE), coronary artery disease (CAD), peripheral artery disease (PAD), atrial fibrillation (AF), ischemic stroke, heart failure (HF), advanced CKD, and all-cause mortality. Advanced CKD was defined as incident stage 4 CKD (N18.4), stage 5 CKD (N18.5), or end-stage kidney disease (N18.6). Because repeated reassignment of CKM stages is not currently feasible using TriNetX, progression to CKM stage 3–4 was operationalized as the occurrence of incident cardiovascular disease or advanced CKD corresponding to advanced CKM stages. Outcome definitions are summarized in Supplementary Table 3. To evaluate biological plausibility and residual confounding, additional analyses were performed using negative control outcomes (hearing loss, age-related cataract, and skin cancer), positive control outcomes (infectious diseases, sepsis, and non-healing wounds), and potential mediating outcomes (elevated C-reactive protein [CRP], atherosclerotic plaque, cardiomegaly, and eGFR < 45 mL/min/1.73 m^2^). Patients were followed for up to 365 days after the index date. Follow-up began on the index date and continued until the first occurrence of the outcome of interest, death, loss to follow-up/end of available records, or completion of 365 days of follow-up, whichever occurred first. The median follow-up duration was 365 days in both groups.

### Propensity score matching

To reduce potential confounding, 1:1 propensity score matching (PSM) was performed using a greedy nearest-neighbor algorithm with a caliper width of 0.1 pooled standard deviations. Matching incorporated all baseline demographic characteristics, comorbidities, medications, and laboratory variables. Covariate balance was assessed using standardized mean differences (SMDs), with an SMD < 0.1 considered indicative of adequate balance (23).

### Statistical analysis

Baseline characteristics were summarized as mean (SD) for continuous variables and number (%) for categorical variables. Time-to-event outcomes were analyzed using Cox proportional hazards models and reported as hazard ratios (HRs) with 95% confidence intervals (CIs). Kaplan–Meier analyses were used to estimate cumulative event incidence, and differences between groups were assessed using the log-rank test. Prespecified subgroup analyses were conducted according to age, sex, CKM stage, obesity, type 2 diabetes, hypertension, dyslipidemia, chronic kidney disease, and malnutrition. To assess the potential impact of unmeasured confounding, E-values were calculated for the primary and secondary outcomes (24). To account for multiple testing, Bonferroni correction was applied across the primary and secondary outcomes, resulting in a significance threshold of *P* < 0.0056 (25). Potential effect modification across subgroups was assessed using the CI overlap method. The availability of baseline laboratory variables is presented in Supplementary Table 7. Missing values were not imputed, and PSM was performed using the data available within the TriNetX platform. Statistical analyses were performed using the built-in analytics functions of the TriNetX platform (TriNetX, Cambridge, MA, USA), including PSM, Kaplan–Meier analyses, Cox proportional hazards models, and subgroup analyses.

### Sensitivity analyses

Several sensitivity analyses were performed to evaluate the robustness of the findings. These included landmark analyses beginning at three months after the index date, analyses restricted to events occurring within the first three months and between three months and one year after the index date, and extended follow-up analyses up to three years. In addition, an alternative exposure definition was evaluated by requiring persistent SZD, defined as a second SZD measurement occurring between 3 and 12 months after the initial SZD measurement.

## Results

### Study population

**FIGURE 1:**
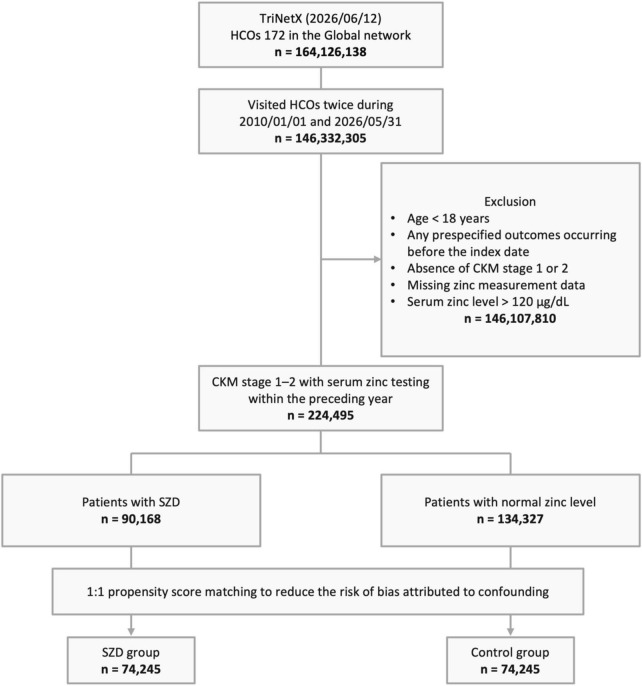
Study cohort process. CKM, cardiovascular-kidney-metabolic; HCOs, healthcare organizations; SZD, serum zinc deficiency.

A total of 146,332,305 individuals had at least two healthcare encounters during the study period. After applying the eligibility criteria, 224,495 patients with CKM stage 1 or 2 who underwent serum zinc testing within the preceding year were identified. Among them, 90,168 patients had SZD, and 134,327 had normal zinc levels. Following 1:1 PSM, 74,245 patients remained in each group (Figure 1). Before matching, patients with SZD had a higher prevalence of comorbidities, including hypertension, type 2 diabetes, chronic kidney disease, anemia, liver disease, and malnutrition. After matching, all baseline characteristics were well balanced, with SMDs below 0.1 except for albumin (Table 1).

**TABLE 1:** Baseline characteristics of the serum zinc deficiency and the control groups before and after matching.

Variables	Before matching			After matching		
	SZD group (*n* = 90,168)	Control group (*n* = 134,327)	Standardized difference	SZD group (*n* = 74,245)	Control group (*n* = 74,245)	Standardized difference
Age, years						
Mean (SD)	50.5 (18.1)	48.4 (17.1)	0.116	49.5 (18.1)	48.9 (17.6)	0.034
Sex, n (%)						
Female	60,264 (67.5)	90,365 (67.9)	0.008	51,587 (69.5)	52,866 (71.2)	0.038
Male	28,933 (32.4)	42,586 (32.0)	0.009	22,592 (30.4)	21,312 (28.7)	0.038
Race, n (%)						
White	57,334 (64.2)	91,016 (68.4)	0.088	48,370 (65.1)	47,876 (64.5)	0.014
Black or African American	13,853 (15.5)	15,430 (11.6)	0.115	10,999 (14.8)	11,686 (15.7)	0.026
Asian	1,834 (2.1)	2,660 (2.0)	0.004	1,480 (2.0)	1,465 (2.0)	0.001
Other race	5,785 (6.5)	8,881 (6.7)	0.008	4,868 (6.6)	4,972 (6.7)	0.006
Unknown race	9,765 (10.9)	14,436 (10.8)	0.003	8,031 (10.8)	7,762 (10.5)	0.012
Comorbidities, n (%)						
Overweight and obesity	22,339 (25.0)	34,060 (25.6)	0.013	19,449 (26.2)	20,510 (27.6)	0.032
Hypertension	25,018 (28.0)	30,386 (22.8)	0.120	18,887 (25.4)	18,876 (25.4)	0
Dyslipidemia	17,706 (19.8)	27,138 (20.4)	0.014	14,585 (19.6)	14,482 (19.5)	0.003
Neoplasms	17,509 (19.6)	18,667 (14.0)	0.150	12,644 (17.0)	12,865 (17.3)	0.008
Type 2 diabetes	12,442 (13.9)	14,468 (10.9)	0.093	9,102 (12.3)	9,042 (12.2)	0.002
Chronic lower respiratory diseases	11,599 (13.0)	13,085 (9.8)	0.100	8,569 (11.5)	8,737 (11.8)	0.007
Anemia	14,689 (16.5)	9,779 (7.3)	0.284	8,459 (11.4)	8,376 (11.3)	0.004
Liver disease	10,010 (11.2)	7,470 (5.6)	0.203	5,634 (7.6)	5,590 (7.5)	0.002
Malnutrition	10,143 (11.4)	5,217 (3.9)	0.283	4,828 (6.5)	4,695 (6.3)	0.007
Nicotine dependence	7,720 (8.6)	5,881 (4.4)	0.172	4,577 (6.2)	4,533 (6.1)	0.002
Chronic kidney disease	5,885 (6.6)	3,918 (2.9)	0.172	3,326 (4.5)	3,254 (4.4)	0.005
Alcohol-related disorders	5,815 (6.5)	2,349 (1.8)	0.240	2,364 (3.2)	2,247 (3.0)	0.009
Medication, n (%)						
Beta-blockers	15,867 (17.8)	15,821 (11.9)	0.166	10,896 (14.7)	10,800 (14.5)	0.004
Diuretics	16,517 (18.5)	14,936 (11.2)	0.206	10,584 (14.3)	10,357 (14.0)	0.009
Statins	9,996 (11.2)	12,437 (9.3)	0.061	7,567 (10.2)	7,500 (10.1)	0.003
Insulin and analogs	11,972 (13.4)	9,203 (6.9)	0.216	7,246 (9.8)	7,164 (9.6)	0.004
Calcium channel blockers	9,797 (11.0)	9,648 (7.2)	0.130	6,840 (9.2)	6,765 (9.1)	0.004
Angiotensin II receptor blockers	6,092 (6.8)	7,222 (5.4)	0.058	4,697 (6.3)	4,633 (6.2)	0.004
ACE inhibitors	6,054 (6.8)	7,491 (5.6)	0.048	4,554 (6.1)	4,545 (6.1)	0.001
Biguanides	4,582 (5.1)	7,269 (5.5)	0.015	3,792 (5.1)	3,721 (5.0)	0.004
GLP1RAs	3,078 (3.4)	4,318 (3.2)	0.011	2,685 (3.6)	2,758 (3.7)	0.005
Sulfonylureas	1,186 (1.3)	1,359 (1.0)	0.029	824 (1.1)	855 (1.2)	0.004
SGLT2is	1,012 (1.1)	1,327 (1.0)	0.013	761 (1.0)	772 (1.0)	0.001
DPP4is	1,114 (1.2)	1,071 (0.8)	0.044	739 (1.0)	734 (1.0)	0.001
Thiazolidinediones	318 (0.4)	360 (0.3)	0.015	237 (0.3)	233 (0.3)	0.001
Alpha-glucosidase inhibitors	130 (0.1)	99 (0.1)	0.021	77 (0.1)	85 (0.1)	0.003
Body mass index, kg/m2						
Mean (SD)	30.2 (9.6)	31.4 (9.2)	0.129	30.9 (9.7)	31.4 (9.4)	0.046
≥ 30	32,005 (35.9)	44,939 (33.8)	0.044	26,859 (36.2)	27,856 (37.5)	0.028
eGFR, mL/min/1.73 m2						
Mean (SD)	90.2 (39.1)	88.3 (29.6)	0.053	90.2 (35.2)	89.1 (33.1)	0.033
< 45	11,380 (12.7)	6,432 (4.8)	0.282	5,900 (7.9)	5,757 (7.8)	0.007
HbA1c, %,						
Mean (SD)	6.0 (1.5)	5.9 (1.2)	0.065	5.9 (1.4)	5.9 (1.3)	0.008
≥ 7	5,320 (6.0)	6,666 (5.0)	0.042	3,928 (5.3)	3,927 (5.3)	0
Albumin, g/dL						
Mean (SD)	3.6 (0.8)	4.1 (0.6)	0.706	3.8 (0.7)	3.9 (0.7)	0.225
< 3.5	28,369 (31.8)	14,254 (10.7)	0.533	14,184 (19.1)	14,098 (19.0)	0.003
Systolic blood pressure, mmHg						
Mean (SD)	123.6 (18.9)	124.9 (17.1)	0.067	124.2 (18.4)	124.9 (17.5)	0.037
≥ 130	52,078 (58.3)	64,964 (48.8)	0.192	40,566 (54.6)	41,394 (55.8)	0.022
Triglyceride, mg/dL						
Mean (SD)	124.1 (107.5)	132.3 (102.9)	0.078	124.5 (90.0)	125.6 (99.7)	0.011
≥ 150	9,775 (10.9)	18,442 (13.9)	0.088	8,276 (11.1)	8,289 (11.2)	0.001
HDL cholesterol, mg/dL						
Mean (SD)	49.7 (20.8)	51.7 (18.5)	0.104	50.8 (20.2)	52.1 (19.2)	0.067
< 40	9,008 (10.1)	12,903 (9.7)	0.013	6,878 (9.3)	6,702 (9.0)	0.008
LDL cholesterol, mg/dL						
Mean (SD)	49.7 (20.8)	51.7 (18.5)	0.104	50.8 (20.2)	52.1 (19.2)	0.067
≥ 160	1,973 (2.2)	4,729 (3.6)	0.080	1,806 (2.4)	1,785 (2.4)	0.002

ACE, angiotensin-converting enzyme; DPP4i, dipeptidyl peptidase 4 inhibitors; eGFR, estimated glomerular filtration rate; GLP1RA, glucagon-like peptide-1 receptor agonist; LDL, low-density lipoprotein; SD, standard deviation; SGLT2i, sodium-glucose co-transporter 2 inhibitor; SZD, serum zinc deficiency.

### Primary outcome

**FIGURE 2:**
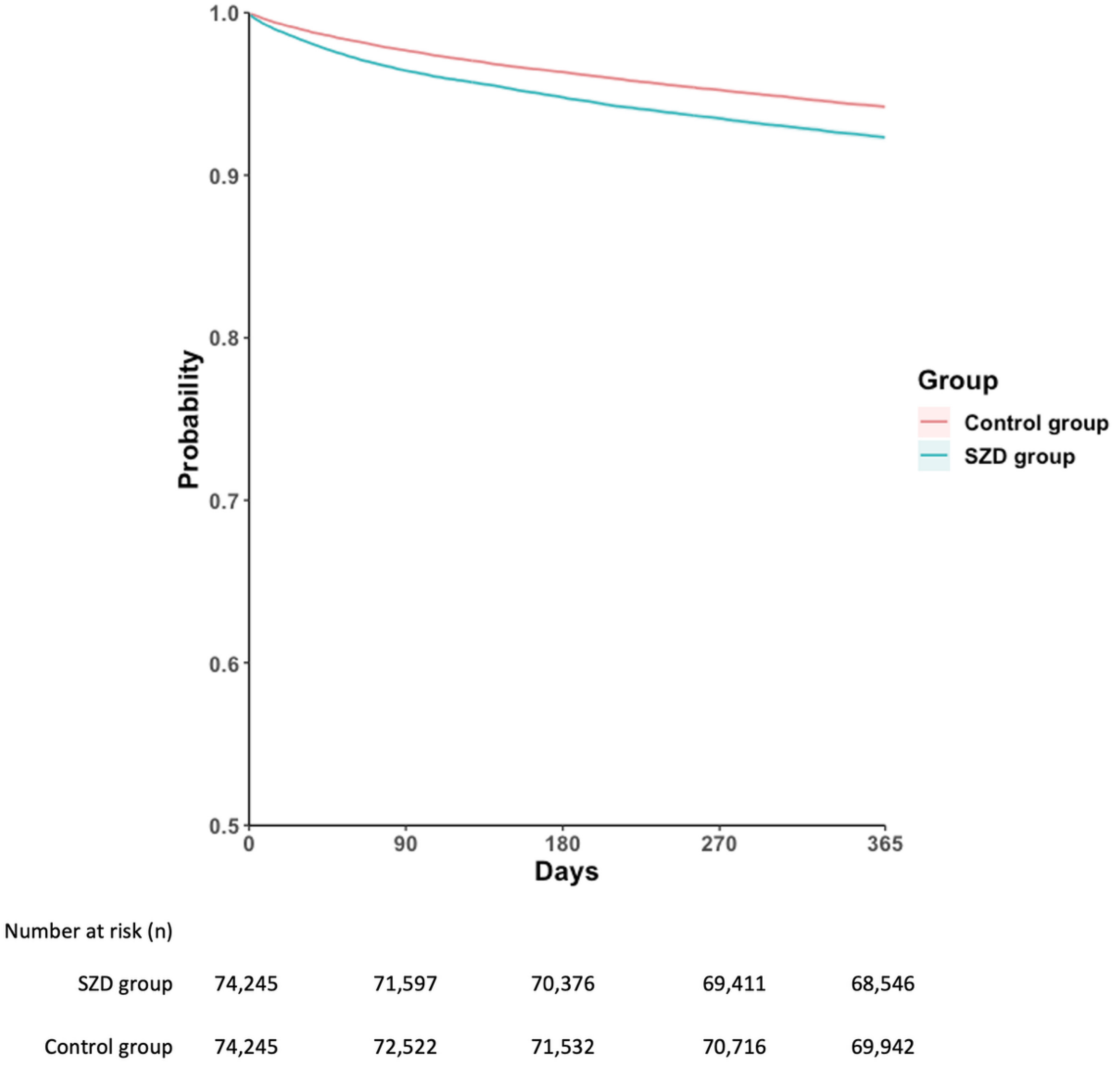
Kaplan–Meier time-to-event free curves of operational progression to CKM stage 3–4 comparison to the serum zinc deficiency and the control groups. The Kaplan–Meier curves depict event-free survival probability for progression to CKM stage 3–4. The *y*-axis represents the probability of remaining free from the outcome rather than cumulative incidence. SZD, serum zinc deficiency.

During follow-up, operational progression from CKM stage 1–2 to stage 3–4 occurred in 4,864 patients (6.6%) in the SZD group and 3,752 patients (5.1%) in the control group. SZD was associated with a significantly increased risk of CKM progression (HR, 1.36; 95% CI, 1.30–1.41; *P* < 0.001), corresponding to an E-value of 2.1 (Table 2). Kaplan–Meier analysis demonstrated a significantly higher cumulative incidence of CKM progression among patients with SZD than among matched controls (Figure 2).

**TABLE 2:** Hazard ratio of outcomes between the serum zinc deficiency and the control groups.

Outcome	SZD group (*n* = 74,245)	Control group (*n* = 74,245)	HR (95% CI)	*P*-value	E-value (95% LCL)
	Events (%)	Events (%)			
Primary outcome					
Operational progression to CKM stage 3–4	4,864 (6.6)	3,752 (5.1)	1.36 (1.30, 1.41)	< 0.001	2.1 (1.9)
Secondary outcomes					
MACE	5,434 (7.3)	4,204 (5.7)	1.35 (1.30, 1.41)	0.001	2.0 (1.9)
CAD	2,731 (3.7)	2,131 (2.9)	1.33 (1.26, 1.41)	< 0.001	2.0 (1.8)
PAD	729 (1.0)	646 (0.9)	1.17 (1.06, 1.31)	0.003	1.6 (1.3)
AF	1,024 (1.4)	748 (1.0)	1.42 (1.29, 1.56)	< 0.001	2.2 (1.9)
Ischemic stroke	638 (0.9)	481 (0.6)	1.38 (1.22, 1.55)	< 0.001	2.1 (1.7)
HF	1,501 (2.0)	971 (1.3)	1.61 (1.48, 1.74)	< 0.001	2.6 (2.3)
Advanced CKD	1,226 (1.7)	955 (1.3)	1.31 (1.21, 1.43)	< 0.001	2.0 (1.7)
All-cause mortality	2,906 (3.9)	1,820 (2.5)	1.65 (1.55, 1.75)	< 0.001	2.7 (2.5)

Bonferroni correction was applied for multiple comparisons across the primary and secondary outcomes. Statistical significance was defined as a two-sided *P*-value < 0.0056. AF, atrial fibrillation; CAD, coronary artery disease; CI, confidence interval; CKD, chronic kidney disease; CKM, cardiovascular-kidney-metabolic; GLP-1 RA, glucagon-like peptide-1 receptor agonist; HF, heart failure; HR, hazard ratio; LCL, lower confidence limit; MACE, major adverse cardiovascular event; PAD, peripheral artery disease; SZD, serum zinc deficiency.

### Secondary outcomes

Compared with the control group, patients with SZD had significantly increased risks of all evaluated secondary outcomes (Table 2). The strongest associations were observed for all-cause mortality (HR, 1.65; 95% CI, 1.55–1.75), heart failure (HR, 1.61; 95% CI, 1.48–1.74), and atrial fibrillation (HR, 1.42; 95% CI, 1.29–1.56). SZD was also associated with increased risks of MACE (HR, 1.35; 95% CI, 1.30–1.41), CAD (HR, 1.33; 95% CI, 1.26–1.41), ischemic stroke (HR, 1.38; 95% CI, 1.22–1.55), advanced CKD (HR, 1.31; 95% CI, 1.21–1.43), and PAD (HR, 1.17; 95% CI, 1.06–1.31) (Table 2). All associations remained statistically significant after Bonferroni correction.

### Subgroup analysis

**FIGURE 3:**
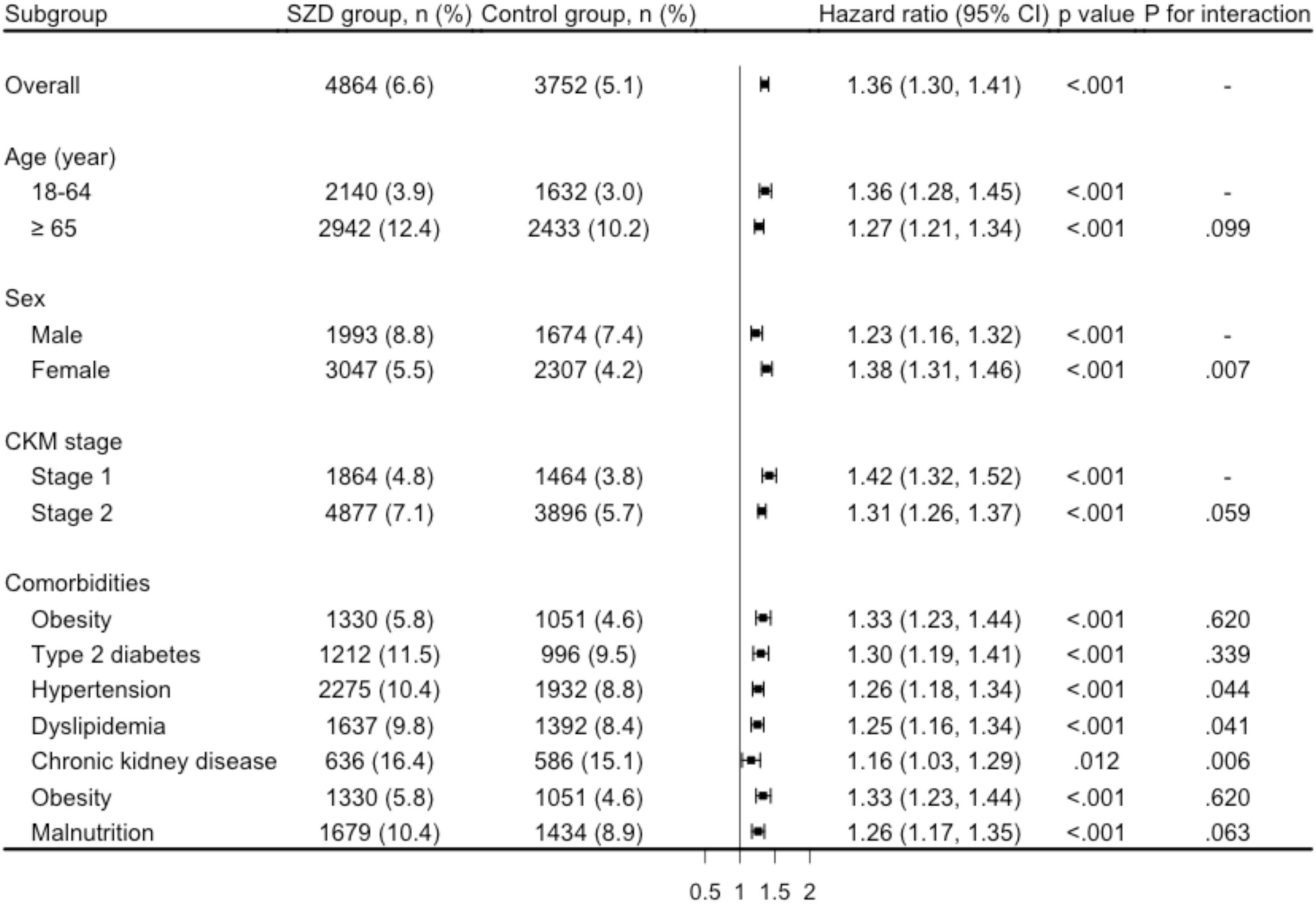
Subgroup analysis for the risk of operational progression to CKM stage 3–4 comparison to the serum zinc deficiency and control groups. Values presented in the final column were derived using the confidence interval overlap method and should be interpreted as exploratory measures of heterogeneity rather than formal interaction tests. CI, confidence interval; CKM, cardiovascular-kidney-metabolic; SZD, serum zinc deficiency.

Subgroup analyses demonstrated generally consistent associations between SZD and progression to CKM stage 3–4 across clinically relevant patient groups (Figure 3).

Potential heterogeneity in the association between SZD and the primary outcome was observed across sex, CKD, hypertension, and dyslipidemia subgroups using the CI overlap method.

### Sensitivity analysis

The association between SZD and CKM progression remained robust across multiple time-related sensitivity analyses (Supplementary Table 5). Although the strongest association was observed during the first 3 months after the index date (HR, 1.57; 95% CI, 1.47–1.66), elevated risks persisted during the 3-month to 1-year period (HR, 1.24; 95% CI, 1.18–1.31) and the 6-month to 1-year period (HR, 1.21; 95% CI, 1.14–1.28). Furthermore, the association remained significant during extended follow-up analyses, including follow-up restricted to events occurring between 1 and 3 years after the index date (HR, 1.12; 95% CI, 1.07–1.16). When persistent SZD was used as an alternative exposure definition, defined as a second SZD measurement occurring 3 to 12 months after the initial measurement, the association remained significant (HR, 1.58; 95% CI, 1.36–1.83; *P* < 0.001) (Supplementary Table 6).

### Negative control, positive control, and potential mediating outcomes

No significant associations were observed between SZD and the negative control outcomes, including hearing loss, age-related cataract, and skin cancer (Supplementary Table 4). In contrast, SZD was associated with increased risks of positive control outcomes, including infectious diseases (HR, 1.24; 95% CI, 1.21–1.26), sepsis (HR, 1.56; 95% CI, 1.47–1.65), and non-healing wounds (HR, 1.31; 95% CI, 1.23–1.40) (Supplementary Table 4). Potential mediating outcomes were also more common among patients with SZD, including elevated CRP levels (HR, 1.27; 95% CI, 1.24–1.31), atherosclerotic plaque (HR, 1.21; 95% CI, 1.09–1.34), cardiomegaly (HR, 1.71; 95% CI, 1.58–1.85), and eGFR below 45 mL/min/1.73 m^2^ (HR, 1.35; 95% CI, 1.31–1.40) (Supplementary Table 4).

## Discussion

In this large multinational PSM cohort study of patients with CKM stage 1–2, SZD was associated with a significantly increased risk of operational progression to CKM stage 3–4. Patients with SZD experienced a 36% higher risk of operational progression to advanced CKM manifestations. SZD was also associated with increased risks of MACEs, CAD, AF, ischemic stroke, HF, advanced CKD, and all-cause mortality. These findings remained consistent across multiple subgroup and sensitivity analyses. Collectively, our findings suggest that SZD may identify individuals at increased risk of adverse CKM trajectories.

To our knowledge, this is among the first large-scale studies to evaluate the association between SZD and disease progression within the recently proposed CKM framework. Previous studies have demonstrated associations between low zinc status and individual cardiovascular, metabolic, and renal disorders. SZD has been linked to insulin resistance, type 2 diabetes, CKD progression, endothelial dysfunction, and increased cardiovascular risk (26–29). However, primarily focused on isolated disease outcomes rather than progression across the integrated CKM continuum. The CKM staging system recognizes that cardiovascular disease, kidney disease, obesity, and metabolic dysfunction represent interconnected manifestations of a shared disease continuum. The present study differs by specifically evaluating operational progression from CKM stage 1–2 to stage 3–4 among individuals with established CKM syndrome among individuals with established CKM stage 1–2, thereby providing a more comprehensive assessment of the potential role of zinc deficiency across interconnected cardiometabolic and renal pathways. Several biological mechanisms may explain the observed association. Zinc plays a critical role in antioxidant defense and regulation of inflammatory pathways. Experimental studies have shown that SZD promotes oxidative stress, activation of proinflammatory cytokines, endothelial dysfunction, and impaired nitric oxide bioavailability (30–32). In addition, zinc is an essential cofactor for numerous enzymes involved in carbohydrate, lipid, and protein metabolism and plays an important role in insulin synthesis, storage, secretion, and signaling. Previous studies have demonstrated that SZD is associated with impaired insulin sensitivity, dysregulated glucose metabolism, adverse lipid profiles, and metabolic dysfunction. These metabolic abnormalities may further accelerate progression across the CKM continuum by promoting obesity, insulin resistance, and cardiometabolic dysfunction. Collectively, these pathophysiological changes contribute to vascular injury, atherosclerosis, insulin resistance, and kidney damage, all of which are central components of CKM progression (33, 34). Consistent with these mechanisms, we observed increased risks of elevated CRP levels, atherosclerotic plaque formation, cardiomegaly, and deterioration of kidney function among individuals with SZD. Although mediation analyses were not performed, these findings support the biological plausibility of the observed associations.

Our positive-control analyses further strengthen the validity of the study findings. SZD was associated with higher risks of infectious diseases, sepsis, and non-healing wounds, outcomes that are well-established clinical consequences of inadequate zinc status. Conversely, no significant associations were observed for hearing loss, age-related cataracts, or skin cancer, which were selected as negative-control outcomes.

The positive-control outcomes were selected because they represent well-recognized clinical manifestations of SZD and therefore support the validity of the exposure classification and the biological plausibility of the study findings. However, these associations may also be influenced by frailty, inflammation, healthcare utilization, and other sources of residual confounding. Consequently, positive-control findings should not be interpreted as evidence excluding residual confounding. In contrast, the absence of associations with negative-control outcomes provides some reassurance against substantial systematic bias. An important finding of this study is the consistent association between SZD and multiple clinically relevant outcomes beyond CKM progression. The strongest associations were observed for HF and all-cause mortality. These findings are noteworthy because HF has recently been recognized as a central manifestation of advanced CKM syndrome. Zinc participates in myocardial energy metabolism, regulation of oxidative stress, and maintenance of cardiomyocyte integrity (35). Therefore, chronic SZD may contribute to adverse cardiac remodeling and increased susceptibility to HF. Similarly, the observed association with advanced CKD is biologically plausible given the established interactions between zinc homeostasis, inflammation, oxidative stress, and renal injury.

From a clinical perspective, serum zinc measurement is an inexpensive and widely available laboratory test that may provide additional information for risk stratification among individuals with CKM stage 1–2. Notably, the association remained significant when persistent SZD was used as an alternative exposure definition, suggesting that sustained SZD may confer an even greater risk than isolated low zinc measurements. Although the present study does not establish causality and should not be interpreted as evidence supporting routine zinc supplementation or screening, it highlights SZD as a clinically relevant risk marker associated with adverse CKM trajectories. Future studies are warranted to determine whether correction of SZD can reduce the risk of CKM progression and improve cardiorenal outcomes. Future studies are warranted to determine whether correction of SZD can reduce the risk of CKM progression and improve cardiorenal outcomes.

Several limitations should be acknowledged. First, the observational design precludes causal inference, and residual confounding cannot be completely excluded despite extensive PSM, E-value analyses, and multiple sensitivity analyses. Second, serum zinc concentrations may be influenced by acute illness, systemic inflammation, nutritional status, and laboratory variability, potentially resulting in exposure misclassification. Although PSM incorporated albumin, malnutrition, anemia, liver disease, and other proxies of nutritional status, residual imbalance remained for albumin as a continuous variable after matching. Furthermore, detailed inflammatory markers and indicators of disease severity were not uniformly available for all participants. Therefore, residual confounding related to nutritional status, inflammation, or illness severity cannot be completely excluded. However, clinically relevant hypoalbuminemia and malnutrition were well balanced after matching, and the association between SZD and the primary outcome remained consistent across the malnutrition subgroup analysis. Third, detailed dietary assessment data were unavailable within the TriNetX database. Information regarding food group consumption, total energy intake, dietary patterns, and macro- and micronutrient intake could not be evaluated. Although BMI, albumin, and malnutrition diagnoses were incorporated as proxies of nutritional status, these variables cannot substitute for comprehensive dietary assessment. Consequently, SZD may reflect broader nutritional inadequacy rather than isolated zinc deficiency, and residual confounding related to overall dietary quality or deficiencies of other nutrients cannot be excluded. Fourth, the primary outcome was defined using an operational CKM staging algorithm derived from electronic health records and may be subject to misclassification. Because repeated reassignment of CKM stages is not directly available within the TriNetX platform, the primary endpoint should be interpreted as operational progression to advanced CKM manifestations rather than direct longitudinal CKM stage transition. Fifth, although several potential mechanistic outcomes were evaluated, formal mediation analyses were not performed; therefore, causal pathways underlying the observed associations could not be established. Sixth, formal competing-risk analyses could not be performed because such methods are not currently available within the TriNetX platform. Given the higher mortality observed among patients with SZD, competing-risk effects may have influenced the estimated risks of nonfatal outcomes. Future studies using datasets that support Fine-Gray or cause-specific hazard models are needed to validate these findings. Seventh, formal exposure-by-subgroup interaction testing was not available within the TriNetX platform. Therefore, potential heterogeneity was assessed using the CI overlap method, and these findings should be interpreted as exploratory rather than definitive evidence of effect modification. Finally, inclusion was restricted to patients who underwent serum zinc testing in routine clinical practice. Because zinc testing is frequently performed in individuals with suspected nutritional deficiencies, chronic illness, inflammation, or other high-risk clinical conditions, selection bias and indication-for-testing bias may have occurred. Although extensive PSM was performed, residual confounding related to the clinical decision to obtain zinc measurements cannot be completely excluded.

## Data Availability

The original contributions presented in this study are included in this article/Supplementary material, further inquiries can be directed to the corresponding authors.
