# Supplementary material for: Serum zinc deficiency and progression of cardiovascular- kidney-metabolic syndrome
Source: Front Nutr. 2026 Jul 23;13:1910233. doi: 10.3389/fnut.2026.1910233 (PMC13441776; doi:10.3389/fnut.2026.1910233)
Supplement: Supplementary file 1 [file Table_1.docx]

**Table S1.** Demographic, diagnostic, procedural, medication, visit, and laboratory codes utilized in the definition of CKM.

| **Category** | **Code** | **Description** |
| --- | --- | --- |
| **CKM stage 1** | | |
| laboratory | UMLS:LNC:39156-5 | Body Mass Index (at least 25.00 kg/m2) |
| laboratory | UMLS:LNC:8280-0 | Waist Circumference at umbilicus by Tape measure (at least 88.00 cm) |
| laboratory | UMLS:LNC:56115-9 | Waist Circumference by NCFS (at least 88.00 cm) |
| laboratory | TNX:9037 | Hemoglobin A1c/Hemoglobin.total in Blood (at least 5.70 %) |
| laboratory | UMLS:LNC:1558-6 | Fasting glucose [Mass/volume] in Serum or Plasma (at least 100.00 mg/dL) |
| diagnosis | UMLS:ICD10CM:N18 | Chronic kidney disease (CKD) |
| diagnosis | UMLS:ICD10CM:E11 | Type 2 diabetes mellitus |
| medication | NLM:ATC:A10A | INSULINS AND ANALOGUES |
| medication | NLM:ATC:A10B | BLOOD GLUCOSE LOWERING DRUGS, EXCL. INSULINS |
| laboratory | TNX:9037 | Hemoglobin A1c/Hemoglobin.total in Blood (at least 6.40 %) |
| **CKM stage 2 (CKD/TG135/HTN/DM)** | | |
| diagnosis | UMLS:ICD10CM:N18 | Chronic kidney disease (CKD) |
| laboratory | TNX:9004 | Triglyceride [Mass/volume] in Serum, Plasma or Blood (at least 135.00 mg/dL) |
| diagnosis | UMLS:ICD10CM:I10 | Essential (primary) hypertension |
| laboratory | TNX:9085 | Blood Pressure, Systolic (at least 130.00 mm[Hg]) |
| laboratory | TNX:9086 | Blood Pressure, Diastolic (at least 80.00 mm[Hg]) |
| medication | NLM:ATC:C02 | ANTIHYPERTENSIVES |
| diagnosis | UMLS:ICD10CM:E11 | Type 2 diabetes mellitus |
| laboratory | TNX:9037 | Hemoglobin A1c/Hemoglobin.total in Blood (at least 6.40 %) |
| medication | NLM:ATC:A10A | INSULINS AND ANALOGUES |
| medication | NLM:ATC:A10B | BLOOD GLUCOSE LOWERING DRUGS, EXCL. INSULINS |
| **CKM stage 2 (WC+HDL+TG)** | | |
| laboratory | UMLS:LNC:8280-0 | Waist Circumference at umbilicus by Tape measure (at least 94.00 cm) |
| laboratory | UMLS:LNC:56115-9 | Waist Circumference by NCFS (at least 94.00 cm) |
| laboratory | TNX:9001 | Cholesterol in HDL [Mass/volume] in Serum or Plasma (at most 40.00 mg/dL) |
| laboratory | TNX:9004 | Triglyceride [Mass/volume] in Serum, Plasma or Blood (at least 150.00 mg/dL) |
| **CKM stage 2 (WC+HDL+SBP)** | | |
| laboratory | UMLS:LNC:8280-0 | Waist Circumference at umbilicus by Tape measure (at least 94.00 cm) |
| laboratory | UMLS:LNC:56115-9 | Waist Circumference by NCFS (at least 94.00 cm) |
| laboratory | TNX:9001 | Cholesterol in HDL [Mass/volume] in Serum or Plasma (at most 40.00 mg/dL) |
| medication | NLM:ATC:C02 | ANTIHYPERTENSIVES |
| laboratory | TNX:9085 | Blood Pressure, Systolic (at least 130.00 mm[Hg]) |
| laboratory | TNX:9086 | Blood Pressure, Diastolic (at least 80.00 mm[Hg]) |
| **CKM stage 2 (WC+HDL+FPG)** | | |
| laboratory | UMLS:LNC:8280-0 | Waist Circumference at umbilicus by Tape measure (at least 94.00 cm) |
| laboratory | UMLS:LNC:56115-9 | Waist Circumference by NCFS (at least 94.00 cm) |
| laboratory | TNX:9001 | Cholesterol in HDL [Mass/volume] in Serum or Plasma (at most 40.00 mg/dL) |
| laboratory | UMLS:LNC:1558-6 | Fasting glucose [Mass/volume] in Serum or Plasma (at least 100.00 mg/dL) |
| **CKM stage 2 (HDL+TG+SBP)** | | |
| laboratory | TNX:9001 | Cholesterol in HDL [Mass/volume] in Serum or Plasma (at most 40.00 mg/dL) |
| laboratory | TNX:9004 | Triglyceride [Mass/volume] in Serum, Plasma or Blood (at least 150.00 mg/dL) |
| medication | NLM:ATC:C02 | ANTIHYPERTENSIVES |
| laboratory | TNX:9086 | Blood Pressure, Diastolic (at least 80.00 mm[Hg]) |
| laboratory | TNX:9085 | Blood Pressure, Systolic (at least 130.00 mm[Hg]) |
| **CKM stage 2 (HDL+TG+FPG)** | | |
| laboratory | TNX:9001 | Cholesterol in HDL [Mass/volume] in Serum or Plasma (at most 40.00 mg/dL) |
| laboratory | TNX:9004 | Triglyceride [Mass/volume] in Serum, Plasma or Blood (at least 150.00 mg/dL) |
| laboratory | UMLS:LNC:1558-6 | Fasting glucose [Mass/volume] in Serum or Plasma (at least 100.00 mg/dL) |
| **CKM stage 2 (TG+FPG+SBP)** | | |
| laboratory | TNX:9004 | Triglyceride [Mass/volume] in Serum, Plasma or Blood (at least 150.00 mg/dL) |
| laboratory | UMLS:LNC:1558-6 | Fasting glucose [Mass/volume] in Serum or Plasma (at least 100.00 mg/dL) |
| medication | NLM:ATC:C02 | ANTIHYPERTENSIVES |
| laboratory | TNX:9086 | Blood Pressure, Diastolic (at least 80.00 mm[Hg]) |
| laboratory | TNX:9085 | Blood Pressure, Systolic (at least 130.00 mm[Hg]) |
| **Zinc status** | | |
| laboratory | TNX:LG23959-6 | Zinc [Mass/volume] in Serum, Plasma or Blood (at most 0.70 ug/mL) |

**Table S2.** Definitions of covariates coding used in this study.

| **Code** | **Description** |
| --- | --- |
| AI | Age at Index |
| 2106-3 | White |
| UNK | Unknown Race |
| F | Female |
| 2054-5 | Black or African American |
| M | Male |
| 2131-1 | Other Race |
| 2028-9 | Asian |
| F17 | Nicotine dependence |
| F10 | Alcohol related disorders |
| E40-E46 | Malnutrition |
| E11 | Type 2 diabetes mellitus |
| I10 | Essential (primary) hypertension |
| E78 | Disorders of lipoprotein metabolism and other lipidemias |
| N18 | Chronic kidney disease (CKD) |
| K76 | Other diseases of liver |
| C00-D49 | Neoplasms |
| J40-J4A | Chronic lower respiratory diseases |
| E66 | Overweight and obesity |
| D64 | Other anemias |
| A10A | INSULINS AND ANALOGUES |
| A10BA | Biguanides |
| A10BB | Sulfonylureas |
| A10BF | Alpha glucosidase inhibitors |
| A10BG | Thiazolidinediones |
| A10BH | Dipeptidyl peptidase 4 (DPP-4) inhibitors |
| A10BJ | Glucagon-like peptide-1 (GLP-1) analogues |
| A10BK | Sodium-glucose co-transporter 2 (SGLT2) inhibitors |
| CV100 | BETA BLOCKERS/RELATED |
| CV200 | CALCIUM CHANNEL BLOCKERS |
| CV700 | DIURETICS |
| CV800 | ACE INHIBITORS |
| CV805 | ANGIOTENSIN II INHIBITOR |
| C10AA | HMG CoA reductase inhibitors |
| 9083 | BMI |
| 9037 | Hemoglobin A1c/Hemoglobin.total in Blood |
| 9004 | Triglyceride [Mass/volume] in Serum, Plasma or Blood |
| 9085 | Blood Pressure, Systolic |
| 9001 | Cholesterol in HDL [Mass/volume] in Serum or Plasma |
| 9002 | Cholesterol in LDL [Mass/volume] in Serum or Plasma |
| 8001 | Glomerular filtration rate/1.73 sq M.predicted [Volume Rate/Area] in Serum, Plasma or Blood by Creatinine-based formula (MDRD) |
| 9045 | Albumin [Mass/volume] in Serum, Plasma or Blood |

**Table S3.** Definitions of outcomes coding used in this study.

| **Code** | **Description** |
| --- | --- |
| **Progression to CKM stage 3-4** | |
| UMLS:ICD10CM:I50 | Heart failure |
| UMLS:ICD10CM:I63 | Cerebral infarction |
| UMLS:ICD10CM:I21 | Acute myocardial infarction |
| UMLS:ICD10CM:I25.9 | Chronic ischemic heart disease, unspecified |
| UMLS:ICD10CM:I48.91 | Unspecified atrial fibrillation |
| UMLS:ICD10CM:I70.2 | Atherosclerosis of native arteries of the extremities |
| UMLS:ICD10CM:N18.5 | Chronic kidney disease, stage 5 |
| UMLS:ICD10CM:N18.6 | End stage renal disease |
| UMLS:ICD10CM:N18.4 | Chronic kidney disease, stage 4 (severe) |
| **MACE** | |
| UMLS:ICD10CM:I63 | Cerebral infarction |
| UMLS:ICD10CM:I21 | Acute myocardial infarction |
| UMLS:ICD10CM:I22 | Subsequent ST elevation (STEMI) and non-ST elevation (NSTEMI) myocardial infarction |
| UMLS:ICD10CM:I46 | Cardiac arrest |
| **CAD** | |
| UMLS:ICD10CM:I21 | Acute myocardial infarction |
| UMLS:ICD10CM:I25.9 | Chronic ischemic heart disease, unspecified |
| **PAD** | |
| UMLS:ICD10CM:I70.2 | Atherosclerosis of native arteries of the extremities |
| **AF** | |
| UMLS:ICD10CM:I48.91 | Unspecified atrial fibrillation |
| **Ischemic stroke** | |
| UMLS:ICD10CM:I63 | Cerebral infarction |
| **HF** | |
| UMLS:ICD10CM:I50 | Heart failure |
| **Advanced CKD** | |
| UMLS:ICD10CM:N18.5 | Chronic kidney disease, stage 5 |
| UMLS:ICD10CM:N18.6 | End stage renal disease |
| UMLS:ICD10CM:N18.4 | Chronic kidney disease, stage 4 (severe) |
| **All-cause mortality** | |
| Deceased | Deceased |
| UMLS:ICD10CM:R99 | Ill-defined and unknown cause of mortality |

**Table S4**. Negative, positive, and potential mediating outcomes associated with serum zinc deficiency.

| Outcome | HR (95% CI) | *P* value |
| --- | --- | --- |
|  |  |  |
| Negative outcomes |  |  |
| Hearing loss | 1.06 (0.98,1.16) | 0.150 |
| Age-related cataract | 1.04 (0.97,1.13) | 0.251 |
| Skin cancer | 1.10 (0.97,1.25) | 0.145 |
| Positive outcomes |  |  |
| Certain infectious and parasitic diseases | 1.24 (1.21,1.26) | <.001 |
| Sepsis | 1.56 (1.47,1.65) | <.001 |
| Non-healing wound | 1.31 (1.23,1.40) | <.001 |
| Potential mediating outcomes |  |  |
| CRP ≥ 5 mg/L | 1.27 (1.24,1.31) | <.001 |
| Atherosclerotic Plaque | 1.21 (1.09,1.34) | <.001 |
| Cardiomegaly | 1.71 (1.58,1.85) | <.001 |
| eGFR < 45 mL/min/1.73 m² | 1.35 (1.31,1.40) | <.001 |

**Table S5**. Time-related sensitivity analyses of the primary outcome

| Outcome | HR (95% CI) | *P* value |
| --- | --- | --- |
|  |  |  |
| Follow-up period from 1-day to 3-months | 1.57 (1.47,1.66) | <.001 |
| Follow-up period from 3-months to 1-year | 1.24 (1.18,1.31) | <.001 |
| Follow-up period from 6-months to 1-year | 1.21 (1.14,1.28) | <.001 |
| Extended follow-up period from 1-day to 3-years | 1.25 (1.21,1.29) | <.001 |
| Extended follow-up period from 1-month to 3-years | 1.16 (1.11,1.21) | <.001 |
| Extended follow-up period from 3-months to 3-years | 1.19 (1.15,1.23) | <.001 |
| Extended follow-up period from 1-year to 3-years | 1.12 (1.07,1.16) | <.001 |

**Table S6**. Sensitivity analysis using persistent serum zinc deficiency as the exposure definition for the primary outcome

| Outcome | HR (95% CI) | *P* value |
| --- | --- | --- |
|  |  |  |
| Progression to CKM stage 3–4 | 1.58 (1.36,1.83) | <.001 |

**Table S7**. Availability of baseline laboratory and clinical variables before propensity score matching

| **Variable** | **SZD group (n=90,168)**  **Available n (%)** | **Control group (n=134,327)**  **Available n (%)** |
| --- | --- | --- |
| Systolic blood pressure | 71,693 (80.3) | 92,935 (69.8) |
| Body mass index | 63,718 (71.4) | 82,659 (62.1) |
| eGFR | 64,258 (72.0) | 83,242 (62.5) |
| Albumin | 58,053 (65.0) | 69,290 (52.1) |
| Triglycerides | 33,960 (38.0) | 55,464 (41.7) |
| Hemoglobin A1c | 31,498 (35.3) | 48,559 (36.5) |
| HDL cholesterol | 29,716 (33.3) | 52,477 (39.4) |
| LDL cholesterol | 29,389 (32.9) | 51,438 (38.6) |
